# Personality, mental health and demographic correlates of hoarding behaviours in a midlife sample

**DOI:** 10.7717/peerj.2826

**Published:** 2016-12-22

**Authors:** Janet K. Spittlehouse, Esther Vierck, John F. Pearson, Peter R. Joyce

**Affiliations:** 1 Department of Psychological Medicine, University of Otago, Christchurch, Christchurch, New Zealand; 2 Biostatistics and Computational Biology Unit, University of Otago, Christchurch, Christchurch, New Zealand

**Keywords:** Personality, TCI, Hoarding, SI-R, Depression

## Abstract

We describe the Temperament and Character Inventory personality traits, demographic features, physical and mental health variables associated with hoarding behaviour in a random community sample of midlife participants in New Zealand. A sample of 404 midlife participants was recruited to a study of ageing. To assess hoarding behaviours participants completed the Savings Inventory-Revised (SI-R), personality was assessed by the Temperament and Character Inventory and self-reported health was measured by the Short Form-36v2 (SF-36v2). Other measures were used to assess socio-demographic variables and current mental disorders. Participants were split into four groups by SI-R total score (scores: 0–4, 5–30, 31–41 and >41). Those who scored >41 on the SI-R were classified as having pathological hoarding. Trend tests were calculated across the four hoarding groups for socio-demographic, personality, mental and physical health variables. SI-R scores ranged from 0 to 58. The prevalence of pathological hoarding was 2.5% and a further 4% reported sub-clinical symptoms of hoarding. Higher hoarding behaviour scores were related to higher Temperament and Character Inventory scores for Harm Avoidance and lower scores for Self-directedness. Persistence and Cooperativeness scores were lower too but to a lesser extent. Trend analysis revealed that those with higher hoarding behaviour scores were more likely to be single, female, unemployed, receive income support, have a lower socio-economic status, lower household income and have poorer self-reported mental health scores. Current depression rates were considerably higher in the pathological hoarding group. Increasing SI-R hoarding behaviour scores were associated with higher scores of negative affect (Harm Avoidance) and lower scores of autonomy (Self-directedness). Those with pathological hoarding or sub-clinical symptoms of hoarding also reported widespread mental and socio-economic problems. In this study it is clear to see the physical, mental and socio-economic problems experienced by those achieving the highest hoarding scores. The prevalence of pathological hoarding was 2.5%, similar to the prevalence reported by other studies. The personality traits associated with hoarding behaviours are discussed.

## Introduction

There has been considerable interest in hoarding behaviour over the last two decades. The Diagnostic and Statistical Manual of Mental Disorders, Fourth Edition (DSM-IV) (American Psychiatric Association, 1994) considered hoarding as a symptom of obsessive compulsive personality disorder and, in extreme cases, as a feature of obsessive compulsive disorder (OCD). In 2013 the American Psychiatric Association released the Diagnostic and Statistical Manual of Mental Disorders, Fifth Edition (DSM-5) (American Psychiatric Association, 2013). A new chapter for obsessive-compulsive related disorders was created and featured four new disorders including hoarding disorder. Until recently there was considerable variation in how researchers defined hoarding (Pertusa et al., 2010). Frost & Hartl’s 1996 working definition is widely accepted and is very similar to the DSM-5 criteria: persistent difficulty throwing away possessions regardless of their value, a need to save the items and distress associated with throwing things away, cluttered living areas to the extent that they can no longer be used for their intended purpose and significant distress or impairment because of the hoarding behaviours (American Psychiatric Association, 2013).

Prevalence estimates for the new classification of hoarding disorder are between two and six percent (American Psychiatric Association, 2013). Very few studies have investigated the prevalence and correlates of hoarding behaviours in a random community sample, and most studies use clinical samples. Results from a random community sample in Germany (Mueller et al., 2009) suggest a prevalence of compulsive hoarding of 4.6% and a study in the UK using DSM-5 criteria found a prevalence of 1.5% for hoarding disorder (Nordsletten et al., 2013). However, hoarding behaviours probably exist on a continuum, and there is little, if any evidence, to validate the boundaries of hoarding disorder as currently defined by the DSM-5.

Recent research has found hoarding to co-occur with other mental disorders including depression, bipolar disorder and personality disorders (Nordsletten et al., 2013; Samuels et al., 2008). Apart from comorbidity with other mental disorders, an epidemiological study carried out in the UK (Nordsletten et al., 2013) found that those with hoarding disorder had a greater likelihood of having a significant physical impairment, being out of work, being older, being single and were more likely to claim benefits. A US study found that hoarding prevalence was inversely related to household income and that people who hoard were more likely to have alcohol dependence than those that do not hoard (Samuels et al., 2008). Results for gender differences in hoarding are conflicting with one community-based study reporting more men (Samuels et al., 2008) and others reporting no gender differences (Bulli et al., 2014; Nordsletten et al., 2013; Tortella-Feliu et al., 2006).

Regarding personality characteristics of hoarding, one study has investigated hoarding and personality variables according to Gray’s personality model (Tortella-Feliu et al., 2006). They found that total hoarding scores, measured by the validated Savings Inventory-Revised (SI-R) (Frost, Steketee & Grisham, 2004), were significantly predicted by sensitivity to reward and sensitivity to punishment when depressive symptoms were controlled for. Further, the SI-R sub-scales of difficulty discarding and clutter were predicted by sensitivity to punishment while acquisition was predicted by sensitivity to reward (Tortella-Feliu et al., 2006). Another study (Fullana et al., 2004) also found that, in OCD patients with hoarding symptoms, high scores on a hoarding symptom scale were positively correlated to sensitivity to punishment and negatively correlated to Eysenck’s Psychoticism scale. Research using the five factor model of personality has found that hoarding is associated with lower scores on conscientiousness, higher neuroticism scores (Hezel & Hooley, 2014; LaSalle-Ricci et al., 2006) and that hoarding severity is predicted by less conscientiousness and more extraversion (LaSalle-Ricci et al., 2006).

Cloninger’s Temperament and Character Inventory (TCI) (Cloninger et al., 1994) has been widely used in research of mental disorders (Fassino et al., 2013; Hansenne et al., 1999). A recent paper (Fassino et al., 2013) has discussed the ‘personality core’ of mental illness and concluded that high Harm Avoidance (which is similar to Gray’s sensitivity to punishment) and low Self-directedness occur in many mental disorders and may represent such a core feature. Several studies (Alonso et al., 2008; Kim, Kang & Kim, 2009; Kusunoki et al., 2000; Lyoo et al., 2003) have described the TCI characteristics associated with OCD which are high Harm Avoidance, low Self-directedness, low Cooperativeness and possibly low Reward Dependence and Novelty Seeking. Two of these papers (Alonso et al., 2008; Kim, Kang & Kim, 2009) have reported TCI associations with hoarding as a symptom of OCD. Using a small sample Alonso (Alonso et al., 2008) found a correlation between scores on a hoarding dimension and high Harm Avoidance scores. However the low numbers reporting hoarding symptoms in this study may have limited the detection of significant relationships between variables. Another study (Kim, Kang & Kim, 2009) found that lower Self-directedness scores and higher Persistence scores predicted a higher hoarding score but this study was restricted by the measure they used to assess hoarding which asks only two questions; one question each about hoarding obsessions and compulsions. To date, there aren’t any studies that describe the associations between hoarding behaviours and TCI personality traits.

From the evidence above, it is clear that pathological hoarding is a debilitating illness and causes significant distress. However, the majority of research into personality and hoarding has been in clinical OCD samples and the assessment of hoarding has been limited to two brief questions. It is important to understand personality variables associated with hoarding because identification of personality traits specific to pathological hoarding may help with understanding the nature of hoarding and may give insight for targets of treatment for a disorder that is hard to treat. Therefore, the aim of this paper is to describe the associations of TCI personality traits, demographic features and physical and mental health with hoarding behaviours.

## Material and Methods

### Study population

Participants were randomly selected from the New Zealand electoral rolls for a study of ageing; the Canterbury Health, Ageing and Lifecourse (CHALICE) study (Schluter et al., 2013). The research was planned as a longitudinal study of the key determinants of health and well-being from midlife onwards. The eligibility criteria were; being 49–51 years of age, an intention to live in the Canterbury area for six of the next 12 months, be living in the community (not an institution) and to be able to complete a four to five hour assessment (for example, have proficient English language skills). Recruitment was from August 2010–October 2013 and coincided with a destructive earthquake sequence that hit the Canterbury area. During this time there were 500 earthquakes of Richter magnitude of four or more beginning with a 7.1 in magnitude that occurred on 4 September 2010. The 6.3 magnitude earthquake in February 2011 resulted in the loss of 185 lives and the seismic activity has resulted in most of the central city having to be demolished.

There are two electoral rolls administrated by the New Zealand government, one Māori roll and a general roll. Māori are the indigenous people of New Zealand. Registration on one of the rolls is compulsory and enrolment statistics from 2012 estimate that 97.1% of 50–54 year olds were registered to vote in the Christchurch City Council area (Electoral Commission Te Kaitiaki Taki Kowhiri, 2013). In New Zealand, 14.6% of the population identify as Māori. In the Canterbury region Māori make up 8% of the population therefore this group were oversampled in the CHALICE study to reflect the wider demographic make-up of the country. CHALICE study participants were considered Māori if they identified Māori as one of the ethnic group or groups that they belong to.

### Assessment

Participants were invited for a half day assessment (four to five hours) at the CHALICE assessment centre. The assessment included personal and family health history, lifestyle questions (e.g., smoking, alcohol consumption, questions about diet and exercise) participant attitudes and opinions about areas of their life (e.g., job satisfaction, coping skills and purpose in life), physical health measures (e.g., height, weight, blood pressure, echocardiogram etc.), cognitive tests and a mental health assessment including personality and hoarding. Mental health, including current major depressive episode, was evaluated using the Mini-International Neuropsychiatric Interview (MINI) (Sheehan et al., 1998). Drinking habits were assesses by the Alcohol Use Disorders Identification Test (AUDIT) (Saunders et al., 1993) and hazardous drinking was defined as achieving a score of eight or more. Further details about CHALICE methods can be found elsewhere (Schluter et al., 2013). To save time on the assessment day, participants were asked to complete some questionnaires, including the Short Form 36v2 (Ware Jr & Sherbourne, 1992) and Cloninger’s short form TCI in the week before the assessment.

### Demographic variables

Demographic information was also collected and socio-economic status was assessed using the Economic Living Standard Index Short Form (ELSI_SF_) (Jensen, Spittal & Krishnan, 2005), developed for use in New Zealand. The ELSI_SF_ asks about ownership restrictions (affordability of basic and luxury items), restriction in social activities because of cost and economising (Jensen, Spittal & Krishnan, 2005). A total score is derived from all the items on the survey. ELSI_SF_ scores range from 0–31. For the analyses in this paper participants were split into three groups; scores of 0–16 (low standard of living, hardship), scores of 17–24 (medium standard of living, comfortable) and scores of 25 or above (socio-economically good or very good). The ELSI_SF_ has excellent internal consistency (coefficient alpha of 0.88) and correlates moderately with other measures associated with standard of living. For household income participants were grouped as follows: low (less than NZ$5,000–50,000), medium (NZ$50,001–100,000) and high (NZ$100,001–150,001 or more). Participants were categorised as receiving income support if they currently received any benefits including in work payments and tax credits. For employment status, full and part time workers, students and those not looking for work were considered employed. Those looking for work or too ill to work were considered unemployed.

### Measures

#### Temperament and Character Inventory (TCI)

The TCI is a personality inventory that is thought to measure seven basic personality traits or domains. These seven domains comprise two aspects of personality; temperament and character. The temperaments are Novelty Seeking, Harm Avoidance, Reward Dependence and Persistence. They are said to develop early in life and are emotional responses to the environment. Novelty Seeking refers to an individuals’ exploratory activity and response to danger with high scorers described as inquisitive and challenge seeking while low scores indicate orderliness, tolerance and indifference. Harm Avoidance measures anxiety and worry. People with high scores on Harm Avoidance may present as fearful, doubtful, timid and easily fatigued as opposed to low scorers who may appear relaxed, self-assured, daring and vigorous. Reward Dependence is how the individual responds to reward, especially social approval, and high scorers are warm, attached and have a need to please others as opposed to cold, independent and objective. Persistence is a measure of perseverance in the face of obstacles and people scoring high on this temperament are described as being diligent, hard-working and perfectionist whereas low scorers are susceptible to fatigue, give up easily and may under achieve.

The character aspect of the TCI measures three dimensions that are conscious, cognitive processes of self-concept that are assumed to mature with age. Self-directedness is a measure of self-determination, with high scorers described as effective, reliable and conscientious. Cooperativeness is the degree to which the individual relates to others. A high score indicates empathy and tolerance. Self-transcendence is the extent to which an individual experiences spiritual ideas and high scores on this trait are said to represent patience and creativity.

As well as the seven traits mentioned above, each temperament has four sub-scales while the character traits have five except Self-transcendence which has three sub-scales. The TCI has a few versions the most current of which is the TCI-R (Cloninger, 2004) and consists of 235 items plus five validity items. To reduce participant burden we used the TCI-140 (136 items and four validity items) of which the seven domains correlate highly with their TCI-R equivalents with values ranging from 0.93 to 0.98 (Farmer & Goldberg, 2008). In the current sample, internal consistency was good with Cronbach’s alpha ranging from 0.80 (Cooperativeness) to 0.89 (Self-transcendence), except for Novelty Seeking which had a Cronbach’s alpha of 0.70.

#### Savings Inventory-Revised (SI-R)

A widely used and valid measure (Frost & Hristova, 2011) of hoarding behaviour is the SI-R (Frost, Steketee & Grisham, 2004). It is a 23 item self-report assessment of hoarding which asks about the three aspects of hoarding behaviour; difficulty discarding, clutter and excessive acquisition. The 23 items of the questionnaire are scored to give a total score and three sub-scale scores that reflect the three behaviours mentioned above. The inventory is suitable for use in both clinical and non-clinical populations (Frost, Steketee & Grisham, 2004).

The DSM-5 criteria for hoarding disorder (American Psychiatric Association, 2013) is difficulty and distress with throwing things away, clutter that prevents using parts of the house for their intended purpose and distress or impairment because of the hoarding behaviours. The SI-R captures these criteria and also provides further information with the excessive acquisition scale. Excessive acquisition is one of the specifiers for hoarding disorder in DSM-5 (American Psychiatric Association, 2013). Each item has a five item Likert scale response format with scores ranging from 0–4. A higher score indicates more hoarding behaviours and possible scores range from 0–92. A total score is derived by adding up the three sub-scale scores. A score over 41 indicates pathological hoarding (Frost & Hristova, 2011) while a score of 30 or over suggests hoarding behaviours that are not necessarily clinically significant. The SI-R has been shown to have good internal consistency and test-retest reliability with a correlation of 0.86 for SI-R total score (Frost, Steketee & Grisham, 2004). The Cronbach’s alpha for the current study was 0.89.

For this study, a time frame was included, participants were asked about hoarding behaviour over the last month. To save time, participants were screened for hoarding behaviour. The screen consisted of four questions from the SI-R: to what extent do you have difficulties throwing things away; to what extent do you have so many things that your house is cluttered; how often do you avoid trying to discard possessions because it is too stressful or time consuming; and how distressed or uncomfortable have you been if you could not acquire something you wanted. If the participant scored two or more for any of the four items (indicating moderate, considerable or severe problems) then they went on to complete the remaining 19 items of the questionnaire.

#### Short Form 36v2 (SF-36v2)

The SF-36v2 is a 36 item questionnaire that measures self-reported health related quality of life. It is considered the ‘gold standard’ in self-reported physical and mental functioning. The first question of the SF-36v2 is a global rating of health and all but one of the 36 items contribute to eight multi-item subscales of health: physical functioning, role limitations due to physical problems (role-physical), bodily pain, general health perception, vitality, social functioning, role limitations due to emotional problems (role-emotional) and mental health. These eight sub-scales are then transformed to provide two summary measures: physical and mental health status. The summary scores are assumed to have a mean of 50 and standard deviation of 10, based on US general population data (Ware et al., 2007).

The first question of the SF-36v2 is a global assessment of health and asks “in general would you say that your health is.” Answers are on a five point Likert scale which are: excellent, very good, good, fair or poor. In this study the global assessment of health was dichotomised into those with good, fair or poor health (low global health status) and those with very good or excellent health.

The questionnaire has high internal consistency with Cronbach’s alpha coefficients of 0.95 for the physical component summary (PCS) and 0.93 for the mental component summary (MCS). Good test retest reliability has been established with intraclass correlation coefficients of 0.87 (PCS) and 0.59 (MCS) across two weeks. The concurrent validity estimates for the 8 subscales are between 0.76 (role-emotional) to 0.93 (general health and mental health) (Ware et al., 2007). In this sample Cronbach’s alpha ranged from 0.80 (social functioning) to 0.94 (role-physical). The SF-36 measures eight of the most commonly used health concepts (Ware et al., 2007); however, it does not measure other concepts such as sleep and cognitive function.

### Statistical methods

Data from the study were stored in a secure database (Progeny Software, Needham, South Norfolk, UK). For data analyses and graphing data were transferred to R 2.4.1 (R Foundation for Statistical Computing, Vienna, Austria) and the coin package (Zeileis et al., 2008). Reporting of method and results were informed by the STROBE guideline (Vandenbroucke et al., 2007).

Four participants did not complete the TCI and one participant did not complete the SF-36v2. All missing data points of the TCI, SF-36v2 and ELSI_SF_ were estimated using the guidelines from the appropriate user manual (Cloninger et al., 1994; Jensen, Spittal & Krishnan, 2005; Ware et al., 2007). There were six respondents with one TCI item missing, one with two missing items and one respondent had five missing items, two of which were from the Harm Avoidance scale. For the validity items, four participants had their TCI data omitted from the analyses because they incorrectly completed three or more validity items. Eight participants missed one item from the SF-36v2 and one participant had two items of missing data. There was one item of missing data for the ELSI_SF_ and for the SI-R on the difficulty discarding/saving subscale, a score for which was calculated from the mean score of the rest of that subscale.

For the analyses the highly skewed, nonlinear hoarding scores were grouped and treated as an ordinal factor. This approach was chosen because of the expected small sample sizes that had pathological hoarding or subclinical pathological hoarding. Rather than using a dichotomous definition of hoarding, the groups allowed exploration of the subclinical features of hoarding behaviour and the dimensional nature of hoarding. Participants were split into the four groups by SI-R total score: The categorical demographic variables were tested for independence with permutation tests (Hothorn & Hornik, 2006) which are equivalent to Cochran Armitage tests for the dichotomous variables, and are robust to distributional assumptions. TCI and SF36v2 scores were treated as continuous with trends tested by linear regression of mean values at the median of each hoarding group. Visual inspection of the data show that, where associations were significant, linear trends were reasonable approximations, hence linear trends were tested in all cases. Mean group differences were calculated using Independent samples *t*-test for TCI and SF36v2 variables showing significant trends across the groups.

(1) No hoarding (group one), had a score of 0–4, most in this group screened negative for hoarding behaviours.
(2) Slight hoarding (group two) achieved a score of 5–30 and reported some hoarding behaviours that were unlikely to be of clinical significance.
(3) Subclinical hoarding (group three) scored 31–41 which was one standard deviation or less than the pathological hoarding cut off score of 41.
(4) Pathological hoarding (group four) were defined as those who scored over 41. 

### Ethics

The Upper South A Regional Ethics Committee granted the CHALICE study ethical approval on the 14 June 2010 (reference: URA/10/03/021). All participants gave written, informed consent for the study. The study complied with the ethical standards for human experimentation as defined by the Helsinki Declaration 1964 (sixth revision 2008).

## Results

### Participants

**Figure 1:**
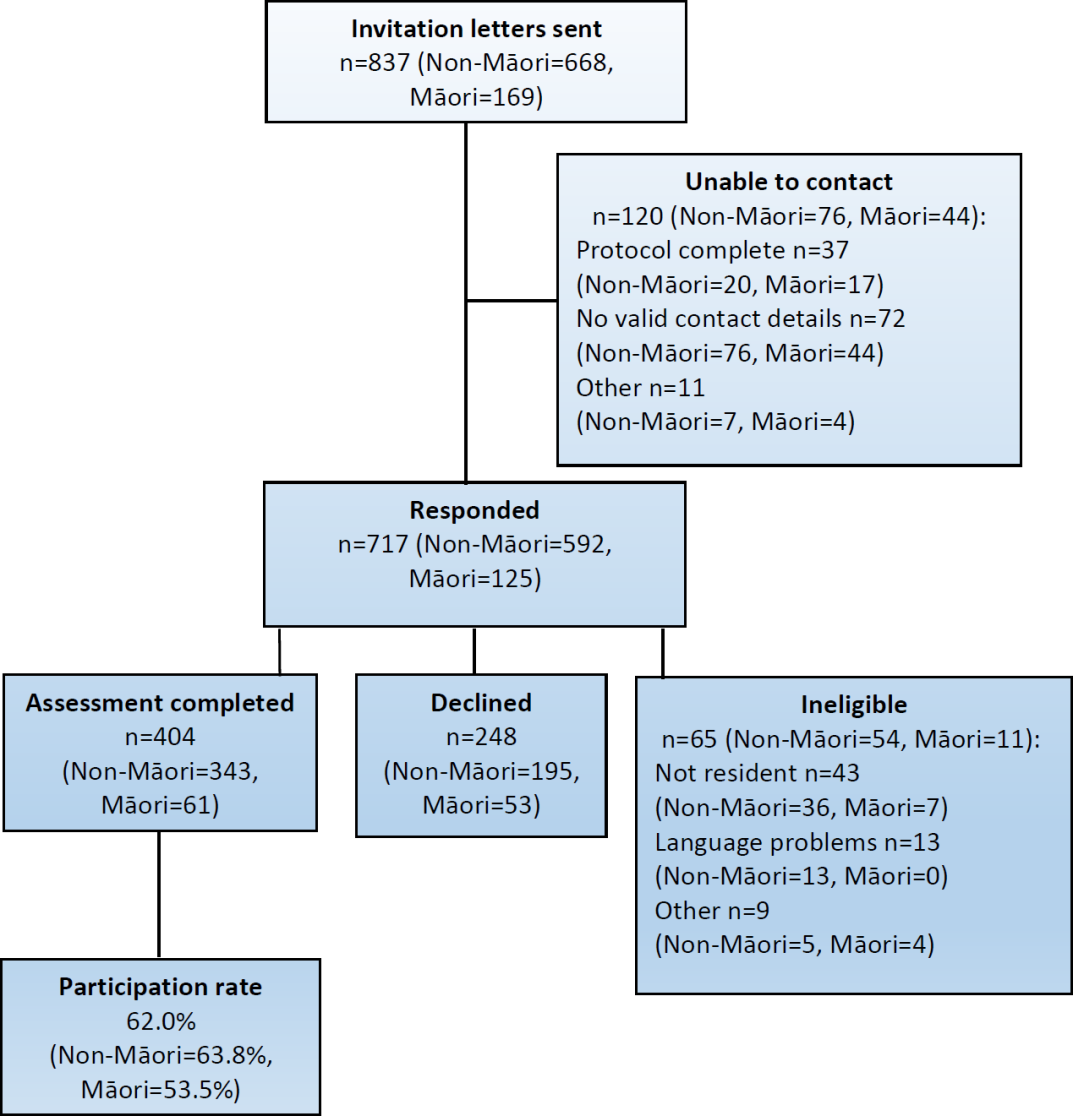
Participant flow for the CHALICE study.

The participation rate for the CHALICE study was 62% with 404 participants completing the assessment (Fig. 1). The participants identifying as Māori were 15.1% (*n* = 61). Table 1 shows that 53.2% of the sample were female, 23% were single and 6.2% were unemployed. For the socio-economic indicators, 19.6% were in receipt of income support, 62.4% described their socio-economic status as high and 44% had a high household income. Compared to New Zealand 2006 census data (Statistics New Zealand, 2013) for the Canterbury region the demographic characteristics of the CHALICE study participants show that in the CHALICE group there was a slightly higher percentage of females (53.2% compared to 50.7%) and 50% of people in the CHALICE sample had a post-secondary qualification or university degree compared to 41% in the census population. The marital status of CHALICE participants were very similar to the census data. For socio-economic status the CHALICE group percentages in three groups was analogous to national data from New Zealand Health Survey 2006/7 (age group 15 years and above) (Ministry of Health, 2008). Comparable data for household income were not available.

In the CHALICE sample 7.9% were assessed as having current depression and 3.0% had OCD. Of the 12 participants with OCD, four had pathological hoarding (33%) and of the ten in the pathological hoarding group, four had OCD (44%). Regarding global health, 43.7% described their health as good, fair or poor as opposed to very good or excellent.

### Hoarding scores distribution

**Figure 2:**
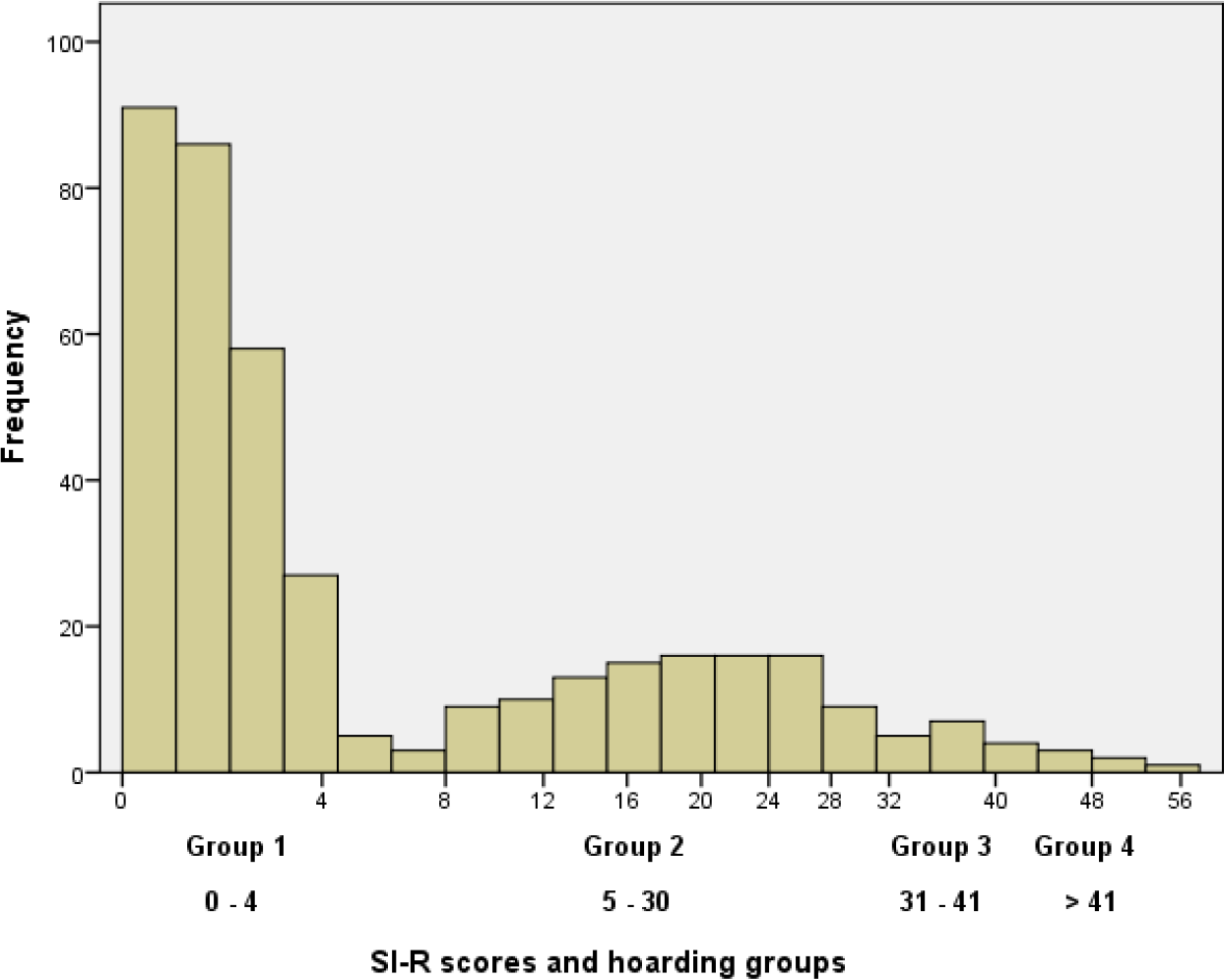
Distribution of SI-R scores for all CHALICE study participants.

Of the 404 participants, 265 screened negative for hoarding behaviour and 139 completed all 23 items of the SI-R. There were three participants who screened positive but had a total SI-R score of four or less and they were included in group one for the analyses. The highly skewed distribution of the scores for the total sample are shown in Fig. 2. Approximately two thirds of the sample reported no or minimal symptoms of hoarding. Among the other third, who completed the full SI-R, the mean score was 21.3 (standard deviation = 11.3). Using predefined cut points, nine (2.5%) scored >41 and 16 (4%) scored 31–41. Total SI-R scores ranged from 0 to 58.

**Table 1:** Demographics for CHALICE study participants by hoarding score group.

	Hoarding (SI-R grouped scores)											
	Group 1 No hoarding (0–4)^a^		Group 2 Slight hoarding (5–30)^a^		Group 3 Subclinical (31–41)		Group 4 Pathological (>41)		Total		Trend	
	*n* = 268		*n* = 110		*n* = 16		*n* = 10		*N* = 404			
**Demographics*** N* %												
Female	145	54.1%	50	45.5%	12	75.0%	8	80.0%	215	53.2%	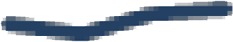	^*^
Single	62	23.1%	17	15.5%	7	43.8%	7	70.0%	215	23.0%	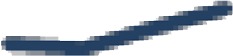	^***^
Unemployed	16	6.0%	3	2.7%	2	12.5%	4	40.0%	356	6.2%	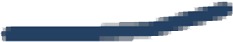	^***^
Income support	49	18.3%	20	18.3%	3	18.8%	7	70.0%	79	19.6%	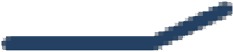	^***^
Socio-economic status												^***^
Low	14	5.2%	8	7.3%	3	18.8%	5	50.0%	30	7.4%	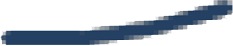	
Medium	80	29.9%	32	29.1%	6	37.5%	4	40.0%	122	30.2%	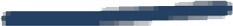	
High	174	64.9%	70	63.6%	7	43.8%	1	10.0%	252	62.4%	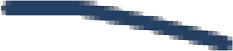	
Household income												^*^
Low	47	18.1%	14	13.3%	4	25.0%	5	50.0%	70	17.9%	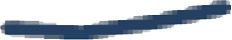	
Medium	93	35.9%	43	41.0%	7	43.8%	4	40.0%	147	37.7%	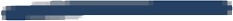	
High	119	45.9%	48	45.7%	5	31.3%	1	10.0%	173	44.4%	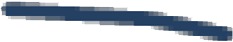	
Low global health	102	38.2%	54	49.1%	11	68.8%	9	90.0%	176	43.7%	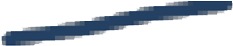	^***^
MDE current	13	4.5%	13	11.8%	2	12.5%	5	50.0%	32	7.9%	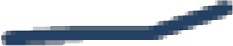	^***^

**Notes.**

Trend shows sparklines by grouped scores with *p* value from asymptotic general independence tests.

* *p* < 0.05,

** *p* < 0.01,

*** *p* < 0.001.

a 1 missing record for income support and low global health, 13 missing records for household income.

MDE Current Major Depressive Episode

### Trends across hoarding groups

Table 1 shows the basic demographic features, general health status and current major depression status by hoarding group. The sparklines show the trends across the groups. The prevalence for pathological hoarding, those scoring >41 on the SI-R, was 2.5% and a further 4% (*n* = 16, SI-R score 31–41) reported sub-clinical symptoms of hoarding. Across hoarding groups there were highly significant trends (*p* <0.001) showing an association of hoarding with being single, unemployed, being in receipt of income support, lower socio-economic status, lower global health status and current major depression. Being female and having a lower household income showed weaker but still significant trends (*p* < 0.05).

Table 2 summarises the SF-36v2 physical and mental summary scores and seven TCI domain scores by hoarding group. There were significant trends in the mean scores for the SF-36v2 mental summary score (*p* < 0.05), these scores decreased as hoarding behaviour scores increased. No significant trends were seen for the physical summary score however, this score trended down nine points between groups three and four. For the TCI personality variables, Harm Avoidance scores trended up (*p* < 0.01) group by group as hoarding behaviour scores increased whereas Self-directedness scores trended down (*p* < 0.01). Cooperativeness and Persistence trended down as hoarding behaviour increased but to a lesser degree (*p* < 0.05). There was no significant trend for Self-transcendence across all groups but the mean scores went up by 9.4 points between groups two and group three.

**Table 2:** Health and personality scores for CHALICE study participants by hoarding score group.

	Hoarding (SI-R grouped scores)											
	Group 1 No hoarding (0–4)		Group 2 Slight hoarding (5–30)		Group 3 Subclinical (31–41)		Group 4 Pathological (>41)		Total		Trend	
	*n* = 268^a^		*n* = 110^b^		*n* = 16		*n* = 10^b^		*N* = 404			
**Health (SF-36v2)**	Mean	(SD)										
Physical summary	51.8	(7.5)	51.0	(8.4)	53.5	(7.4)	44.5	(9.5)	51.5	(7.8)	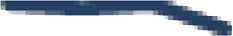	
Mental summary	51.9	(8.8)	48.3	(9.5)	40.9	(9.0)	32.2	(13.2)	50.0	(9.8)	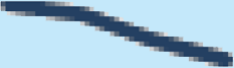	^*^
**Personality (TCI)**												
Novelty seeking	54.5	(7.8)	53.7	(7.7)	59.0	(9.6)	59.3	(9.3)	54.6	(7.9)	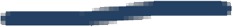	
Harm avoidance	53.8	(11.4)	58.5	(12.6)	66.6	(12.0)	70.7	(14.2)	56.0	(12.4)	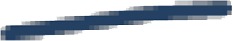	^**^
Reward dependence	67.3	(10.5)	63.5	(9.6)	66.3	(11.1)	64.1	(9.0)	66.2	(10.4)	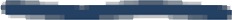	
Persistence	69.7	(10.7)	69.4	(10.4)	66.6	(10.1)	63.4	(8.6)	69.4	(10.6)	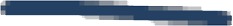	^*^
Self-directedness	77.3	(10.6)	70.8	(10.6)	64.8	(6.7)	56.7	(9.8)	74.6	(11.3)	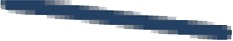	^**^
Cooperativeness	79.0	(8.3)	77.2	(8.9)	76.3	(10.4)	74.1	(6.2)	78.3	(8.6)	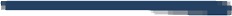	^*^
Self-transcendence	39.9	(11.4)	38.3	(10.9)	47.7	(10.3)	46.9	(13.9)	40.0	(11.4)	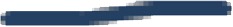	

**Notes.**

Trend shows sparklines by grouped scores with *p* value from linear regression on group medians.

* *p* < 0.05.

** *p* < 0.01.

*** *p* < 0.001.

a 1 missing record for SF36v2, 6 missing for TCI.

b 1 missing record for TCI.

### Mean differences between hoarding groups

Mean Harm Avoidance scores were significantly different between the hoarding groups except for groups three and four. The mean difference between groups one and two was −4.7 (95% CIs [−7.3 to −2.0]) and for groups two and three it was −8.1 (95% CIs [−14.7 to −1.44]). For Self-directedness means between all groups were significantly different. The mean change for groups one and two was 6.5 (95% CIs [4.1–8.8]), groups two and three was 6.0 (95% CIs [0.6–11.4]) and for groups three and four it was 8.1 (95% CIs [1.3–15.0]). There were no significant differences in means for Persistence and Cooperativeness between the groups.

For the SF-36v2 mental summary scores the significant mean differences were 3.6 (95% CIs [1.6–5.6]) between hoarding groups one and two and 7.4 (95% CIs [2.5–12.5]) between groups two and three. Hoarding groups three and four were not significantly different.

## Discussion

In this study we have examined associations of personality, demographic variables, self-reported physical and mental health with four groups of hoarding behaviour (none, slight, subclinical and pathological) defined by scores on the SI-R.

The distribution of scores was highly skewed with most participants reporting no or slight hoarding behaviours. The hoarding scores were grouped and treated as an ordinal factor so that subclinical features of hoarding behaviour could be explored. The cut points defined by Frost & Hristova (2011) using ROC analyses, appear to just be arbitrary rather than revealing a zone of rarity, a common problem in psychiatric diagnoses (Kendell & Jablensky, 2003). Figure 2 shows that there may be a point of rarity at SI-R scores of 4–8, however, this is probably as a result of using four SI-R questions to screen for hoarding behaviours. The results suggest that hoarding behaviour is on a continuum ranging from no or very little hoarding tendencies to pathological hoarding, rather than a dichotomous diagnostic category as defined by DSM-5 (American Psychiatric Association, 2013).

Using an SI-R cut point of 41 or more, the 2.5% prevalence in this study is higher than the 1.5% prevalence reported in the epidemiological study conducted in the UK (Nordsletten et al., 2013) but lower than other studies (Bulli et al., 2014; Mueller et al., 2009; Samuels et al., 2008). The UK study prevalence for hoarding disorder may be lower than our prevalence of pathological hoarding because it was based on the result of a diagnostic interview for hoarding disorder, a review of self-report questionnaires by experienced researchers and clutter analysis. Bulli et al. (Bulli et al., 2014) found a prevalence of 3.7% to 6% in their two Italian convenience samples. They also used the SI-R with a cut off score of 41 or above and, in one study, they excluded those with a psychiatric disorder which may have resulted in an under estimation of the prevalence. Possible explanations by the authors of their relatively high rates are that the cut off score for the SI-R may not be appropriate across different cultures and further research in Italian samples is needed. Another possibility the authors discuss is that error may have been introduced by using on online version of the SI-R with some of the sample. Mueller et al. (2009) used a revised 19 item version of the SI-R and the prevalence of compulsive hoarding in a random community sample was 4.6% when the SI-R score cut off was 28. They also reported the prevalence using 36 as the cut off score and this estimate was 1.5%. Meaningful comparisons between this and Mueller’s study are difficult because four items of the SI-R were removed and the cut-off points for the SI-R were different but the prevalence reported here lies between the two prevalence estimates for their study (Mueller et al., 2009). A study in North America (Samuels et al., 2008) found a prevalence of 3.7% using one question about hoarding from the DSM-IV (American Psychiatric Association, 1994) criteria for obsessive compulsive disorder. All of the above study participants were of mixed ages but Samuels et al. (2008) reported a prevalence of 2.9% for the 45–54 age group, very similar to the prevalence reported here. The hoarding prevalence results for the CHALICE study suggest that an SI-R score of 41 or above was an appropriate cut point for this sample.

The key TCI personality variables associated with hoarding behaviour were Self-directedness and Harm Avoidance. Self-directedness scores were lower in the groups with higher hoarding behaviour scores and Harm Avoidance scores were higher in these groups. Additionally, higher hoarding behaviour scores were associated with smaller but significant downward trends for Persistence and Cooperativeness.

Significant trends in Harm Avoidance and Self-directedness scores are apparent even when hoarding behaviour symptoms are limited (group two) and mean scores vary significantly group by group with the exception of groups three and four for Harm Avoidance. These trends are also reflected in the SF-36v2 mental summary score which also trended down significantly with each group, except for groups three and four. These group differences suggest that hoarding behaviours are dimensional in nature.

Low Self-directedness and high Harm Avoidance are a core feature of OCD (Alonso et al., 2008; Kim, Kang & Kim, 2009; Kusunoki et al., 2000; Lyoo et al., 2003) and many other mental health disorders (Fassino et al., 2013) so the trends seen here are not surprising. Harm Avoidance is essentially a measure of anxiety, fear and shyness. Hoarding behaviour has been linked with higher rates of anxiety (Bulli et al., 2014; Nordsletten et al., 2013) and worry (Reid et al., 2011). Also, people who hoard have emotional links to objects which may create anxiety and worry about possessions not seen in people who don’t hoard (Frost & Hartl, 1996). Self-directedness is a measure of self-autonomy and high scores represent maturity, responsibility and self-acceptance. Research has shown that people who hoard have a problem with routine decision making, inattention and executive function (Frost & Hartl, 1996; Hall et al., 2013). These characteristics may interfere with aspects of Self-directedness such as behaviour regulation and goal-orientated behaviours and perhaps explain why lower Self-directedness scores were associated with higher hoarding behaviour scores.

In this sample Persistence trended down across hoarding groups. Given associations between Persistence and perfectionism, this finding is not consistent with suggestions that hoarding is associated with perfectionism (Frost & Gross, 1993). This finding is also inconsistent with a study using OCD patients (Kim, Kang & Kim, 2009) where hoarding symptoms were positively correlated with higher Persistence. Alternatively, it is possible that differences in Persistence may be a personality marker of the differences between OCD and pathological hoarding but this finding would need to be replicated before it can be confirmed.

Cooperativeness also trended down as hoarding behaviour increased. Other personality research has shown little difference between population means in measures of agreeableness in those with hoarding symptoms (LaSalle-Ricci et al., 2006). The mean difference between the hoarding disorder group and those with no or very little hoarding behaviours was relatively small in comparison to the differences between these two groups for Harm Avoidance and Self-directedness. Reward Dependence, which is highly correlated with Cooperativeness, showed no overall trend.

Subclinical hoarding behaviour was associated with greater Novelty Seeking and Self-transcendence compared to those with slight hoarding behaviour, although the overall trend was not significant. Those with hoarding disorder had similar scores on these two traits compared to those with subclinical behaviours. It is possible that these two traits are revealing subtle differences between those at very little risk of developing problematic hoarding behaviour and those at risk of, or those who have already developed pathological hoarding. Impulsivity, one of the features of Novelty Seeking, has been found to be increased in those with compulsive hoarding (Grisham et al., 2007). Another study looking at OCD symptom dimensions in OCD patients found that hoarding behaviour was inversely related to Novelty Seeking (Fullana et al., 2004). This suggests that impulsivity may differentiate between those with symptoms of hoarding associated with a primary diagnosis of OCD as opposed to those whose prominent disorder is hoarding. Regarding Self-transcendence, higher scores on this scale have been associated with schizotypy and magical thinking, especially when one or both variables of Self-directedness and/or Cooperativeness are low (Laidlaw et al., 2005). In the CHALICE study, both Self-directedness and Cooperativeness trended down as hoarding behaviour increased, which suggests an immature personality that may be prone to this type of symptom. Furthermore, magical thinking and erroneous beliefs have also been observed in those with hoarding behaviours (Frost & Hartl, 1996; Samuels et al., 2007).

For the demographic variables, higher hoarding behaviour scores were associated with being single, unemployed, receiving income support, being in a lower socio-economic group and, to a lesser degree, being female and having a lower household income. These results are very similar to a recent study (Nordsletten et al., 2013) but different to two other studies (Bulli et al., 2013; Mueller et al., 2009) who found no differences between people who hoard and those that do not for a range of socio-demographic variables. These differences may be explained by differing methodologies. One of the studies (Bulli et al., 2014) used a convenience sample and excluded anyone with psychiatric disorder and the other study (Mueller et al., 2009) had a large representative sample that included a much wider age range than the sample reported here. Many studies report no gender difference but Samuels et al. (2008) reported that the prevalence of hoarding was higher for men than women whereas we found the opposite. In our sample, 80% in the pathological hoarding group were female. It is possible that women were more willing to endorse hoarding behaviours for what is often described as an embarrassing and shameful disorder.

It is clear that for those in the pathological hoarding group economic hardship and impairment of mental and physical functioning is widespread. For example 70% of people in this group were single and/or receiving income support, 40% were unemployed, and 50% had low socio-economic status and/or household income. Only 10% of this group described their health as very good or excellent, 50% were currently depressed and their self-reported mental and physical health scores were comparatively low. For compulsive hoarding a prevalence rate of 40–50% of comorbid depression is not unusual and has been reported elsewhere (Frost, Steketee & Tolin, 2011).

For this 50-year-old sample, those that currently reported subclinical hoarding behaviours (group three) may develop hoarding disorder in future years. Although for many, hoarding behaviours typically start in adolescence (Grisham et al., 2006), they are known to increase with each decade of life (Ayers et al., 2010; Frost et al., 2000). Additionally, for some, pathological hoarding behaviours have a later onset and are associated with stress or loss (Grisham et al., 2006). Approximately a quarter report onset of hoarding disorder after the age of 40 (Dozier, Porter & Ayers, 2016). Loss, for example loss of career, through retirement, or loss of a spouse may be more likely to occur as people get older. In the CHALICE study it is possible that loss associated with the earthquakes (for example loss of your home) will have an impact on hoarding behaviours, especially for those in the subclinical group. For the subclinical group, intervention strategies used sooner rather than later may be more helpful than waiting for the disorder to become established, which is notoriously hard to treat (Steketee & Frost, 2003; Tolin et al., 2010).

There are some limitations with the current study. The restricted age range of the sample may mean that the results are only applicable to this age group. Hoarding groups three and four have low sample numbers meaning findings should be interpreted cautiously and may need validating in an independent cohort. All measures were self-report which are open to perceptual bias and socially desirable responding. However the prevalence of pathological hoarding reported here suggests that bias for the self-report measures are minimal. The data are cross-sectional so causation cannot be established. We did not carry out a home visit or an independent assessment of clutter which may be ideal. However, the SI-R shows strong correlations to other hoarding measures including observer ratings of clutter (Frost, Steketee & Grisham, 2004). It is possible that the TCI personality profile of people with severe hoarding is somewhat different to those seen here and further study of TCI characteristics using a clinical sample may be revealing. The high rate of depression in the pathological hoarding group may have affected TCI ratings at interview (Spittlehouse et al., 2010). However, it is often the case that people with pathological hoarding present with comorbidities, especially depression, so the results here may be a realistic reflection of the personality of people with pathological hoarding. The assessments were carried out during a considerable earthquake sequence that has been shown to affect the self-reported mental health scores of this cohort (Spittlehouse et al., 2014). However, the impact of these experiences on hoarding behaviour is not known but more general research on trauma has shown that traumatic incidents may increase hoarding behaviours in some people (Grisham et al., 2006).

The strengths of the study are that it is a random community sample and the participants were not recruited to a study of hoarding which means that bias from self-selection or convenience samples is not an issue. To our knowledge, this is the first study to look at the TCI characteristics of hoarding behaviour in a community sample, using a specific hoarding questionnaire.

## Conclusions

As hoarding behaviours increased, scores on TCI personality variables Harm Avoidance trended up while Self-directedness, Persistence and Cooperativeness trended down. Those with more hoarding behaviours were more likely to be single, unemployed and have lower scores on socio-economic indicators. Self-reported mental health score was lower in those groups reporting more hoarding behaviours and the trend for the pathological hoarding group suggests that their physical health was impaired. In this study it is clear to see the physical, mental and socio-economic problems experienced by those achieving the highest hoarding scores.

## Additional Information and Declarations

### Data Availability

The following information was supplied regarding data availability: The raw data was made available for review but cannot be published with the article due to the restrictions of the ethics committee, which does not allow data to be shared or used by any people other than those specified in the study protocol.

## References

[ref-1] Alonso P, Menchon JM, Jimenez S, Segalas J, Mataix-Cols D, Jaurrieta N, Labad J, Vallejo J, Cardoner N, Pujol J (2008). Personality dimensions in obsessive-compulsive disorder: relation to clinical variables. Psychiatry Research.

[ref-2] American Psychiatric Association (1994). Diagnostic and statistical manual of mental disorders.

[ref-3] American Psychiatric Association (2013). The diagnostic and statistical manual of mental disorders: DSM 5.

[ref-4] Ayers CR, Saxena S, Golshan S, Wetherell JL (2010). Age at onset and clinical features of late life compulsive hoarding. International Journal of Geriatric Psychiatry.

[ref-5] Bulli F, Melli G, Carraresi C, Stopani E, Pertusa A, Frost RO (2014). Hoarding behaviour in an Italian non-clinical sample. Behavioural and Cognitive Psychotherapy.

[ref-6] Cloninger CR (2004). Feeling good: the science of well-being.

[ref-7] Cloninger CR, Przybeck TR, Svrakic DM, Wetzel RD (1994). The temperament and character inventory (TCI): a guide to its development and use.

[ref-8] Dozier ME, Porter B, Ayers CR (2016). Age of onset and progression of hoarding symptoms in older adults with hoarding disorder. Aging and Mental Health.

[ref-9] Electoral Commission Te Kaitiaki Taki Kowhiri (2013). Enrolment statistics by council: Christchurch city as at 12 March 2013. http://www.elections.org.nz/research-statistics/enrolment-statistics-council?name=Christchurch+City&=Apply.

[ref-10] Farmer RF, Goldberg LR (2008). A psychometric evaluation of the revised Temperament and Character Inventory (TCI-R) and the TCI-140. Psychological Assessment.

[ref-11] Fassino S, Amianto F, Sobrero C, Abbate DG (2013). Does it exist a personality core of mental illness? A systematic review on core psychobiological personality traits in mental disorders. Panminerva Medica.

[ref-12] Frost RO, Gross RC (1993). The hoarding of possessions. Behaviour Research and Therapy.

[ref-13] Frost RO, Hartl TL (1996). A cognitive-behavioral model of compulsive hoarding. Behaviour Research and Therapy.

[ref-14] Frost RO, Hristova V (2011). Assessment of hoarding. Journal of Clinical Psychology.

[ref-15] Frost RO, Steketee G, Grisham J (2004). Measurement of compulsive hoarding: saving inventory-revised. Behaviour Research and Therapy.

[ref-16] Frost RO, Steketee G, Tolin DF (2011). Comorbidity in hoarding disorder. Depression and Anxiety.

[ref-17] Frost RO, Steketee G, Williams LF, Warren R (2000). Mood, personality disorder symptoms and disability in obsessive compulsive hoarders: a comparison with clinical and nonclinical controls. Behaviour Research and Therapy.

[ref-18] Fullana MA, Mataix-Cols D, Caseras X, Alonso P, Manuel Menchón J, Vallejo J, Torrubia R (2004). High sensitivity to punishment and low impulsivity in obsessive-compulsive patients with hoarding symptoms. Psychiatry Research.

[ref-19] Grisham JR, Brown TA, Savage CR, Steketee G, Barlow DH (2007). Neuropsychological impairment associated with compulsive hoarding. Behaviour Research and Therapy.

[ref-20] Grisham JR, Frost RO, Steketee G, Kim HJ, Hood S (2006). Age of onset of compulsive hoarding. Journal of Anxiety Disorders.

[ref-21] Hall BJ, Tolin DF, Frost RO, Steketee G (2013). An exploration of comorbid symptoms and clinical correlates of clinically significant hoarding symptoms. Depression and Anxiety.

[ref-22] Hansenne M, Reggers J, Pinto E, Kjiri K, Ajamier A, Ansseau M (1999). Temperament and character inventory (TCI) and depression. Journal of Psychiatric Research.

[ref-23] Hezel DM, Hooley JM (2014). Creativity, personality, and hoarding behavior. Psychiatry Research.

[ref-24] Hothorn T, Hornik K, Van De Wiel MA, Zeileis A (2006). A Lego system for conditional inference. The American Statistician.

[ref-25] Jensen J, Spittal M, Krishnan V (2005). ELSI short form: user manual for a direct measure of living standards.

[ref-26] Kendell R, Jablensky A (2003). Distinguishing between the validity and utility of psychiatric diagnoses. American Journal of Psychiatry.

[ref-27] Kim SJ, Kang JI, Kim CH (2009). Temperament and character in subjects with obsessive-compulsive disorder. Comprehensive Psychiatry.

[ref-28] Kusunoki K, Sato T, Taga C, Yoshida Y, Komori K, Narita T, Hirano S, Iwata N, Ozaki N (2000). Low novelty seeking differentiates obsessive compulsive disorder from major depression. Acta Psychiatrica Scandinavica.

[ref-29] Laidlaw TM, Dwivedi P, Naito A, Gruzelier JH (2005). Low self-directedness (TCI), mood, schizotypy and hypnotic susceptibility. Personality and Individual Differences.

[ref-30] LaSalle-Ricci VH, Arnkoff DB, Glass CR, Crawley SA, Ronquillo JG, Murphy DL (2006). The hoarding dimension of OCD: psychological comorbidity and the five-factor personality model. Behaviour Research and Therapy.

[ref-31] Lyoo IK, Yoon T, Kang DH, Kwon JS (2003). Patterns of changes in temperament and character inventory scales in subjects with obsessive-compulsive disorder following a 4-month treatment. Acta Psychiatrica Scandinavica.

[ref-32] Ministry of Health (2008). A portrait of health. Key results of the 2006/7 New Zealand health survey.

[ref-33] Mueller A, Mitchell JE, Crosby RD, Glaesmer H, De Zwaan M (2009). The prevalence of compulsive hoarding and its association with compulsive buying in a German population-based sample. Behaviour Research and Therapy.

[ref-34] Nordsletten AE, Reichenberg A, Hatch SL, De la Cruz LF, Pertusa A, Hotopf M, Mataix-Cols D (2013). Epidemiology of hoarding disorder. British Journal of Psychiatry.

[ref-35] Pertusa A, Frost RO, Fullana MA, Samuels J, Steketee G, Tolin D, Saxena S, Leckman JF, Mataix-Cols D (2010). Refining the diagnostic boundaries of compulsive hoarding: a critical review. Clinical Psychology Review.

[ref-36] Reid JM, Arnold E, Rosen S, Mason G, Larson MJ, Murphy TK, Storch EA (2011). Hoarding behaviors among nonclinical elderly adults: correlations with hoarding cognitions, obsessive-compulsive symptoms, and measures of general psychopathology. Journal of Anxiety Disorders.

[ref-37] Samuels JF, Bienvenu OJ, Grados MA, Cullen B, Riddle MA, Liang KY, Eaton WW, Nestadt G (2008). Prevalence and correlates of hoarding behavior in a community-based sample. Behaviour Research and Therapy.

[ref-38] Samuels JF, Bienvenu 3rd OJ, Pinto A, Fyer AJ, McCracken JT, Rauch SL, Murphy DL, Grados MA, Greenberg BD, Knowles JA, Piacentini J, Cannistraro PA, Cullen B, Riddle MA, Rasmussen SA, Pauls DL, Willour VL, Shugart YY, Liang KY, Hoehn-Saric R, Nestadt G (2007). Hoarding in obsessive-compulsive disorder: results from the OCD Collaborative Genetics Study. Behaviour Research and Therapy.

[ref-39] Saunders JB, Aasland OG, Babor TF, De la Fuente JR, Grant M (1993). Development of the Alcohol Use Disorders Identification Test (AUDIT): WHO collaborative project on early detection of persons with harmful alcohol consumption—II. Addiction.

[ref-40] Schluter PJ, Spittlehouse JK, Cameron VA, Chambers S, Gearry R, Jamieson HA, Kennedy M, Lacey CJ, Murdoch DR, Pearson J, Porter R, Richards M, Skidmore PML, Troughton R, Vierck E, Joyce PR (2013). Canterbury Health, Ageing and Life Course (CHALICE) study: rationale, design and methodology. The New Zealand Medical Journal.

[ref-41] Sheehan DV, Lecrubier Y, Sheehan KH, Amorim P, Janavs J, Weiller E, Hergueta T, Baker R, Dunbar GC (1998). The Mini-International Neuropsychiatric Interview (M.I.N.I.): the development and validation of a structured diagnostic psychiatric interview for DSM-IV and ICD-10. The Journal of Clinical Psychiatry.

[ref-42] Spittlehouse JK, Joyce PR, Vierck E, Schluter PJ, Pearson JF (2014). Ongoing adverse mental health impact of the earthquake sequence in Christchurch, New Zealand. Australian and New Zealand Journal of Psychiatry.

[ref-43] Spittlehouse JK, Pearson JF, Luty SE, Mulder RT, Carter JD, McKenzie JM, Joyce PR (2010). Measures of temperament and character are differentially impacted on by depression severity. Journal of Affective Disorders.

[ref-44] Statistics New Zealand (2013). NZStat Census 2006. http://nzdotstat.stats.govt.nz/wbos/Index.aspx.

[ref-45] Steketee G, Frost R (2003). Compulsive hoarding: current status of the research. Clinical Psychology Review.

[ref-46] Tolin DF, Fitch KE, Frost RO, Steketee G (2010). Family informants’ perceptions of insight in compulsive hoarding. Cognitive Therapy and Research.

[ref-47] Tortella-Feliu M, Fullana MA, Caseras X, Andion O, Torrubia R, Mataix-Cols D (2006). Spanish version of the savings inventory-revised: adaptation, psychometric properties, and relationship to personality variables. Behavior Modification.

[ref-48] Vandenbroucke JP, Von Elm E, Altman DG, Gotzsche PC, Mulrow CD, Pocock SJ, Poole C, Schlesselman JJ, Egger M (2007). Strengthening the Reporting of Observational Studies in Epidemiology (STROBE): explanation and elaboration. PLoS Medicine.

[ref-49] Ware Jr JE, Sherbourne CD (1992). The MOS 36-item short-form health survey (SF-36). I. Conceptual framework and item selection. Medical Care.

[ref-50] Ware Jr JE, Kosinski M, Bjorner JB, Turner-Bowker DM, Gandek B, Maruish ME (2007). User’s manual for the SF-36v2 health survey.

[ref-51] Zeileis A, Wiel MA, Hornik K, Hothorn T (2008). Implementing a class of permutation tests: the coin package. Journal of Statistical Software.

